# Soil-transmitted helminthes and *Schistosoma mansoni* infections among primary school children at Ambasame primary school, North-West Ethiopia: a cross-sectional study

**DOI:** 10.1186/s12887-022-03534-5

**Published:** 2022-08-05

**Authors:** Daniel Getacher Feleke, Abdurahaman Ali, Habtye Bisetegn, Habtu Debash, Workineh Birara, Alehegn Andualem

**Affiliations:** 1 Department of Microbiology, Immunology and Parasitology, College of Health Sciences, Addis Ababa University, Addis Ababa, Ethiopia; 2 Department of Medical laboratory Science, College of Medicine and Health Sciences, Wollo University, Dessie, Ethiopia

**Keywords:** Soil transmitted helminthes, *Schistosoma mansoni*, School children, Intestinal parasitic infections, Ethiopia

## Abstract

**Background:**

*Schistosomiasis* and soil-transmitted helminthiasis (STHs) are the major public health problem in the world especially in school age children. Therefore, this study aimed to determine the burden of soil transmitted helminths and *Schistosoma mansoni* among Ambesame primary school children, North-West Ethiopia.

**Method:**

A cross sectional study was carried out at Ambasame primary school children from March to May, 2019. Study participants were selected using systematic random sampling technique. Socio-demographic characteristics and other factors were collected using structured questionnaire. Moreover, stool samples were examined microscopically using wet mount and formol ether concentration techniques. Data were entered and analyzed using SPSS version 20. Logistic regression analysis was done to investigate the association between dependent and independent variables. *P*-value less than 0.05 was considered as statistically significant.

**Result:**

The overall prevalence of intestinal parasites was 117(31.2%). The prevalence of soil-transmitted helminthes and S.*mansoni* was 110 (29.3%) using formol ether concentration technique. The most predominant parasite was *S. mansoni* (10.7%), followed by hookworm (5.6%). Multivariate logistic regression analysis revealed that, helminthic infection was associated with children less than 7 years of age (*P*-value = 0.019, AOR = 3.29, 95% CI (1.21–8.91)); fathers who are able to read and write (*P*-value< 0.001, AOR = 5.4, 95% CI (2.37–12.33)); absence of latrine (*P*-value = 0.016, AOR = 12.96, 95% CI (1.60–104.87)) and untrimmed nail (*P*-value = 0.043, AOR = 2.09, 95% CI (1.02–4.27)).

**Conclusion:**

This study revealed that the prevalence of intestinal helminthes among Ambasame primary school children was relatively high. The lower educational status of father, absence of latrine and untrimmed finger nail showed statistically significant association with intestinal helminthic infection. This indicates the school community, health offices and other stakeholders should plan a strategy to tackle problems associated with sanitary condition. Furthermore, Health policy makers, healthcare workers and health extension workers should enhance their effort of awareness creation for school children, parents, school community about personal hygiene, environmental sanitation, intestinal parasites transmission, prevention and control. Moreover, mass deworming of school children and periodic screening for parasitic infection should be done.

## Introduction

Intestinal parasitic infections are major public health problem worldwide particularly in developing countries [1]. Schistosomiasis and soil-transmitted helminthiasis are among the most common parasitic infections in the world, especially in Sub-Saharan Africa [2, 3]. Soil-transmitted helminthes infected approximately 2 billion people worldwide particularly in tropical countries [4, 5]. Globally an estimated 1.45 billion people are infected with *Ascaris lumbricoides*, 1.3 billion with hookworms and 1.05 with *Trichuris trichiura* [6]. Moreover, about 300 million heavily infected people with helminthes suffer from severe morbidity and more than 150,000 people are died annually [7]. Soil transmitted helminths infection are important cause of morbidity in school age children especially in primary school children [8, 9]. Globally, more than 270 million pre-school children and over 600 million school children in endemic areas need treatment and preventive interventions [10]. Soil-transmitted helminthic infections are usually associated with poverty, lack of sanitation, overpopulation, poor personal and environmental hygiene [11].

Schistosomiasis is also major health problem in the world and it continues to be serious public health concern in developing countries where *S. mansoni* and *S. haematobium* are widespread [12]. School children who are under 15 years of age and living in endemic areas are highly vulnerable for schistosomiasis [13]. People can be exposed to Schistosoma when they are in contact with contaminated water during activities of fishing, farming, swimming, washing, bathing, recreation, and irrigation [14].

In Ethiopia, intestinal parasitic infections are highly endemic and they are the predominant causes of outpatient morbidity due to low living standards, poor environmental sanitation, unsafe human waste disposal systems, lack of safe water supply, and low socio-economic status [15]. Helminthic infections are persist in Ethiopia or elsewhere due to the least attention given to them and the low rank given in the list of priority during public health program planning as their effect is not usually directly measured in terms of mortality figs [16]. Several studies reported the prevalence of Soil-transmitted helminthes and *S. mansoni* conducted in Ethiopia. Among the previous studies reported the prevalence of soil-transmitted helminthes and *S. mansoni,* the prevalence was (66.7%) in North Gondar, (26.9%) in Umolante district, (82.4%) in Zarima town, (35.2%) in Chuahit town, (15%) in Hiruy Abaregawi and 30.5% in Haike primary school children [6, 12, 17–19].

The prevalence varied from 15% to-82.4% in different geographical areas of the country. As per the information from teachers and school directors there is frequent absence of students from class. Moreover, we have noticed there are the known risk factors of intestinal parasites around the school that include unsanitary conditions. However, the prevalence of soil-transmitted helminthic infections and schistosomiasis in the local area has not yet properly documented. This study will help for school community to improve their environmental sanitation and follow their students’ personal hygiene and to health office of Dera district to design periodic mass-deworming program. Therefore, this study aimed to assess prevalence associated risk factors of soil transmitted helminthes and *Schistosoma mansoni* among primary school children at Ambesame primary school, North-West Ethiopia.

## Methods

### Study area

This study was conducted at Ambesame primary school that is found in Dera district, South Gondar zone, Amhara region, Ethiopia. The district is located 535 km northwest of Addis Ababa (the capital city of Ethiopia) and 42 km away from Bahir Dar (Capital city of Amhara region). The altitude and longitude of the town is 11° 45″ N 37° 30″ E and 1500 to 2000 m above the sea level. The average annual rain fall and temperature of the Dera district is 1250 mm and 30 °C. Based on the 2007 national census conducted by the Central Statistical Agency of Ethiopia (CSA), this district has a total population of 248,464, an increase of 17.01% over the 1994 census, of whom 126,961 are men and 121,503 women; 16,772 or 6.75% are urban inhabitants.

### Study design and period

This cross sectional study was conducted from March to May, 2019.

### Source population

All Ambasame primary school community were taken us the source of population in this study.

### Study population

This study included all Ambasame school children who didn’t took any anti-helminthic drugs 2 weeks before the data collection.

### Sample size determination and sampling technique

The sample size was determined by using single population proportion formula using the proportion of school children infected with intestinal parasites from previous study 66.7% [6]. Moreover, marginal error of 5% and estimate with 95% confidence interval were assumed during sample size calculation. The final calculated sample size was 375 including the 10% non-response rate. The sample size was distributed proportional to the total number of students in each grade level. Similarly, the sample size was proportionated to sections of the grade based on their number of students when the grade level has more than one section. Finally, study participants were selected from each grade level and sections using systematic simple random sampling technique.

### Data collection methods and procedure

Pre-tested structured questionnaire was used to assess socio demographic characteristics and intestinal parasite associated risk factors. Approximately*,* about 5 g of fresh stool sample was collected after proper instruction, was given for children with clean, dry, leak proof and labeled stool sample collection cups. The quality of the stool sample was maintained by collecting and processing it according to the standard operating procedures (SOPs). Stool samples collected and checked for sample volume and contamination. Moreover, safety and specimen handling procedures were strictly followed. Wet mount microscopic examination was performed from a portion of the stool sample immediately after collection for detection of eggs and larvae of helminthes, and cysts and trophozoites of protozoan parasites. The remaining part of the stool samples were preserved in 10% formalin solution and transported to Ambasame town health center for formol-ether concentration procedure. After formol-ether concentration procedures, sediment was examined microscopically for parasite detection. Standard operational procedures (SOPs) were strictly followed during formol-ether concentration procedures and microscopic examination of sediments. Microscopic examination of stool samples was performed by senior experienced medical laboratory technologists and technicians. In general, all methods were performed in accordance with the relevant guidelines and regulations and by strictly standard operational procedures (SOPs).

### Data analysis

Data were entered and analyzed using SPSS version 20. Logistic regression analyses were performed to investigate the association between the helminthic infections and associated risk factors. In all comparisons, *P*-value < 0.05 was considered as statistically significant.

### Ethical considerations

Ethical clearance was obtained from the Ethical review committee of Wollo University, College of Medicine and Health Sciences, Department of Medical Laboratory sciences. Permission was obtained from district office. Written informed consents were obtained from the parents/guardians of children after explaining the purpose and the procedures of the study. Study participants who were positive for intestinal parasitic infection were treated with appropriate anti-helminthic drugs.

## Result

### Socio-demographic characteristics of study participants

Of the total 375 school children 206(54.9%) were males. The mean + SD of age of school children was 11.97 + 3.36 years. Majority; 236(62.9%) of the school children were between 7 and 14 years old age (Table 1).

**Table 1:** Socio-demographic characteristics and other risk factors of Anbesame Primary ‘School students from March, 2019- May, 2019

Variables	Category	Number (%)
Age	< 7	38 (10.1)
	7–14	236 (62.9)
	> 14	101 (26.9)
Sex	Male	206 (54.9)
	Female	169 (45.1)
Grade level	Grade 1–4	167 (44.5)
	Grade 5–8	208 (55.5)
Father education	Illiterate	96 (25.6)
	Read & write	36 (9.6)
	Primary school	64 (17.1)
	Secondary school	38 (10.1)
	College and above	141 (37.6)
Mother education	Illiterate	156 (41.6)
	Read and write	15 (4.0)
	Primary school	39 (10.4)
	Secondary school	30 (8.0)
	College	135 (36.0)
Residence	Rural	164 (43.7)
	Urban	211 (56.3)
Water source	Tape water	341 (90.9)
	River	12 (0.3)
	Lake	2 (6.9)
	Well	20 (1.9)

### Prevalence of intestinal helminthes and S.*mansoni*

The overall prevalence of intestinal parasites was 117(31.2%). The overall prevalence of intestinal helminthes and *S. mansoni* was110 (29.3%) using formol ether concentration technique. The overall prevalence of *S. mansoni* was 40(10.7%) with 18(4.8%) and 37(9.8%) prevalence using wet-mount and formol ether concentration technique, respectively. Ten different species of intestinal parasites were detected. The most predominant helminth was *S. mansoni* (10.7%), followed by hookworm (5.6%). The least common helminth was *Strongloides stercoralis* (0.5%). Furthermore, double intestinal parasitic infection was 14 (3.7%) whereas there was only 1 (0.3%) triple infection (Table 2).

**Table 2:** Distribution of intestinal helminthes species among Anbesame Primary ‘School students from March, 2019- May, 2019

Parasites detected	Wet mount	Formol-ether concentration	Total
	Number (%)	Number (%)	Number (%)
Hookworm	7 (1.9)	15 (4)	15 (4)
*Ascaris lumbricoides*	9 (2.4)	9 (2.4)	9 (2.4)
*Enterobius vermicularis*	4 (1.0)	5 (1.3)	5 (1.3)
*Hymenolepis nana*	2 (0.5)	8 (2)	8 (2)
*Taenia spps*	6 (1.5)	8 (2)	8 (2)
*Strongloides sterocoralis*	2 (0.5)	2 (0.5)	2 (0.3)
*Schistosoma mansoni*	18 (4.8)	37 (9.8)	37 (9.8)
*Trichuris trichiura*	7 (1.9)	11 (2.9)	11 (2.9)
*Giardia lamblia*	3 (0.8)	0 (0)	3 (0.8)
*Entamoeba histolytica*	4 (1.0)	0 (0)	4 (1.0)
*Ascaris lumbricoides* and hookworm	1 (0.3)	2 (0.5)	2 (0.5)
*Enterobius vermicularis* and *Hymenolepis nana*	1 (0.3)	1 (0.3)	1 (0.3)
Hookworm and *Hymenolepis nana*	2 (0.5)	1 (0.3)	1 (0.3)
Hookworm and Taenia spp.	1 (0.3)	1 (0.3)	1 (0.3)
Hookworm and *Enterobius vermicularis*	2 (0.5)	2 (0.5)	2 (0.5)
*Schistosoma mansoni* and *Trichuris trichiura*	1 (0.3)	1 (0.3)	1 (0.3)
*Hymenolepis nana* and *Taenia spps*	1 (0.3)	1 (0.3)	1 (0.3)
Hookworm, *Enterobius vermicularis*, *Hymenolepis nana*		1 (0.3)	1 (0.3)
*Ascaris lumbricoides* and *Enterobius vermicularis*		1 (0.3)	1 (0.3)
*Ascaris lumbricoides* and *Taenia spps*		1 (0.3)	1 (0.3)
*Enterobius vermicularis* and *Trichuris trichiura*		1 (0.3)	1 (0.3)
*Schistosoma mansoni* and hookworm		1 (0.3)	1 (0.3)
*Schistosoma mansoni* and *Hymenolepis nana*		1 (0.3)	1 (0.3)
Total	**71 (18.9)**	**110 (29.3)**	**117 (31.2)**

### Analysis of soil-transmitted helminthes and *S. mansoni* associated risk factors

In bivariate analysis; age group of 7–14 years, higher grade level, lower educational status of both mother and father, rural residence, using lake as a water source, absence of latrine, absence hand washing hands after defecation and before meal, unclean nail, untrimmed nail and presence of animals contact were significantly associated (*P*-value < 0.05) with soil transmitted helminthes and *S. mansoni* infections. However, after including risk factors with a *P*-value less than or equal to 0.20 in to multivariate logistic regression model to identify the independent contribution of each risk factors for soil transmitted helminthes and *S. mansoni* infections. The multivariate stepwise logistic analysis showed that, Soil transmitted helminthes and *S. mansoni* infection was independently associated with; age group of less than 7 years, the lower educational status of father; absence of latrine and untrimmed finger nail (Table 3).

**Table 3:** Bivariate and multivariate logistic analysis of factors associated with intestinal helminthes among Anbesame Primary school children from March to May, 2019

Characteristics		Intestinal parasitic infections		COR(95% CI)	***P***-value	AOR(95% CI)	***P***-value
		Positive [N (%)]	Negative [N (%)]				
Age	< 7	16 (4.3)	22 (5.9)	0.74 (0.35–1.58)	0.437	3.29 (1.21–8.91)	**0.019**
	7–14	51 (13.6)	185 (49.3)	0.28 (0.17–0.46)	< 0.001	0.59 (0.31–1.09)	0.093
	> 14	50 (13.3)	51 (13.6)	1		1	
Grade level	Grade 1–4	37 (9.9)	130 (34.7)	1		1	
	Grade 5–8	80 (21.3)	128 (34.1)	2.20 (1.39–3.49)	0.001	1.38 (0.59–3.23)	0.453
Father education	Illiterate	45 (12.0)	51 (13.6)	4.77 (2.60–8.75)	< 0.001	3.68 (1.98–6.91)	**< 0.001**
	Read & write	20 (5.3)	16 (4.3)	6.76 (3.04–15.04)	< 0.001	5.41 (2.37–12.33)	**< 0.001**
	Primary school	24 (6.4)	40 (10.7)	3.25 (1.64–6.41)	0.001	3.13 (1.57–6.21)	**0.001**
	Secondary school	6 (1.6)	32 (8.5)	1.01 (0.38–2.71)	0.978	1.01 (0.38–2.71)	0.982
	College and above	22 (5.9)	119 (31.7)	1		1	
Mother education	Illiterate	73 (19.5)	83 (22.1)	4.78 (2.72–8.37)	< 0.001	0.27 (0.01–7.43)	0.354
	Read & write	7 (1.9)	8 (2.1)	4.75 (1.56–14.50)	0.006	0.28 (0.01–13.11)	0.518
	Primary school	10 (2.7)	29 (7.7)	1.87 (0.80–4.41)	0.151	0.232 (0.01–8.58)	0.427
	Secondary school	6 (1.6)	24 (6.4)	1.36 (0.50–3.72)	0.553	0.94 (0.06–14.84)	0.967
	College	21 (5.6)	114 (30.4)	1		1	
Residence	Rural	78 (20.8)	86 (22.9)	4.00 (2.52–6.36)	< 0.001	1.43 (0.58–3.55)	0.439
	Urban	39 (10.4)	172 (45.9)	1		1	
Water source	Tape water	96 (25.6)	245 (65.3)	1		1	
	River	1 (0.3)	0 (0.0)	–		–	
	Lake	16 (4.3)	10 (2.7)	4.08 (1.79–9.31)	0.001	1.51 (0.59–3.86)	0.386
	Well	4 (1.1)	3 (0.8)	3.40 (0.75–15.49)	0.113	0.97 (0.19–5.05)	0.970
Latrine presence	Yes	107 (28.5)	257 (68.5)	1		1	
	No	10 (2.7)	1 (0.3)	24.01 (3.04–189.96)	0.003	12.96 (1.60–104.87)	**0.016**
Washing hands after toilet	Yes	43 (11.5)	147 (39.2)	1		1	
	No	74 (19.7)	111 (29.6)	2.28 (1.45–3.57)	< 0.001	1.07 (0.60–1.89)	0.819
Washing hands before food	Yes	87 (23.2)	217 (57.9)	1		1	
	No	30 (8.0)	41 (10.9)	1.83 (1.07–3.11)	0.027	0.72 (0.37–1.42)	0.342
Trimmed nail	Yes	95 (25.3)	239 (63.7)	1		1	
	No	22 (5.9)	19 (5.1)	2.91 (1.51–5.63)	0.001	2.09 (1.02–4.27)	**0.043**
Shoe wearing habit	Yes	109 (29.1)	251 (66.9)	1		1	
	No	8 (2.1)	7 (1.9)	0.38 (0.13–1.07)	0.068	0.89 (0.25–3.13)	0.854
Animals contact	Yes	56 (14.9)	182 (48.5)	2.61 (1.66–4.09)	< 0.001	0.89 (0.43–1.84)	0.753
	No	61 (16.3)	76 (20.3)	1		1	

## Discussion

Schistosomiasis and soil-transmitted helminthiasis are among the most common parasitic infections in the world, especially in Sub-Saharan Africa [2, 3].

The present study assessed the prevalence of intestinal helminthic infections in school children in north western Ethiopia. The results of this study showed the occurrence of several intestinal helminthes of public health importance among school children. The overall prevalence of geohelminthes and *S. mansoni* infection was 31.2% (117/375). *Schistosoma mansoni* was the dominant parasite detected in this study (34.2%, 40/117).

The prevalence of intestinal helminthes in this study was higher than reports from Southern India (7.8%), Nigeria (25.6%) and Southern Ethiopia (26.9%) [12, 20, 21]. The difference could be associated with variations in the study area and study seasons, socio-economic status of participants and socio-demographic characteristics and availability of lake near to the school could contribute for the higher prevalence of soil-transmitted helminthes and *S. mansosni* in the present study. On the other hand, the prevalence of geohelminthes and *S. mansoni* infection in the present study was found to be lower than findings from Nigeria (46.3%), Congo (52.9%), Kenya (86%), Nigeria (54.70%) [22–25] and studies conducted in Ethiopia; Gorgora and Chuahit (66.7%), Zarima town (82.4%) and Adwa town (63%) [6, 19, 26].

Furthermore, the finding of this study had comparable prevalence with the 30.5% prevalence reported from North-Eastern Ethiopian study [26]. Generally, the difference in prevalence might be associated with variations in study season and the period where awareness educations and health promotion activities that are being conducted by medias to reduce the prevalence of communicable diseases and the presence of governmental attention to implement neglected tropical disease prevention and control programs could attributed for reduced prevalence intestinal helminthes and *S. mansoni* in the present study. Moreover, the lower prevalence of soil transmitted helminthes and *S. mansoni* due to the method of diagnosis used in this study which were wet mount and formol-ether concentration technique. In contrast to this some of the previous studies were used Kato-Katz technique for the detection of intestinal helminth.

The prevalence of *S. mansoni* infection in this study was found to be 34.2% (40/117) which is slightly in line with a study reported from Gorgora and Chuahit towns, North-West Ethiopia (33.7%) [6]. On the other hand it was lower than findings of Adwa Town 63% [26] and Haik, North eastern Ethiopia (76.5%) [27]. The difference from these studies was due to variations from time period; study area, socio-demographic characteristics and differences in utilization of species specific concentration techniques; like Kato-Katz technique for *S.mansoni*. The prevalence of soil-transmitted helminthes and *S.mansoni* was significantly higher among children less than 7 years of age (*P*-value = 0.019, AOR = 3.29, 95% CI (1.21–8.91)); fathers who are able to read and write (*P*-value< 0.001, AOR = 5.4, 95% CI (2.37–12.33)); absence of latrine (*P*-value = 0.016, AOR = 12.96, 95% CI (1.60–104.87)) and untrimmed nail (*P*-value = 0.043, AOR = 2.09, 95% CI (1.02–4.27)) which was in line with a report from Southern India [20]. This findings were in line with the fact that many of the schools in Ethiopia are not fulfill the minimum standard and they lacks facilities such as latrine, clean water and appropriate waste disposal system. Moreover, it’s obvious that the educational status of students’ parent, their personal hygiene and age could expose them for soil-transmitted helminthes.

## Conclusion

This study revealed that the prevalence of intestinal helminthes among Ambasame primary school children was relatively high. The lower educational status of father, absence of latrine and untrimmed finger nail showed statistically significant association with intestinal helminthic infection. This indicates the school community, health offices and other stakeholders should plan a strategy to tackle problems associated with sanitary condition. Furthermore, Health policy makers, healthcare workers and health extension workers should enhance their effort of awareness creation for school children, parents, school community about personal hygiene, environmental sanitation, intestinal parasites transmission, prevention and control. Moreover, mass deworming of school children should be screened for parasitic infection periodically. In this study Kato-Katz technique was not performed due to resource limitation which is more sensitivity for parasite detection and to estimate the intensity of *S. mansoni* and other helminthes infection.

## Data Availability

The authors confirm that all data underlying the findings are fully available without restriction. All relevant data are within the manuscript.
